# PDGF controls contact inhibition of locomotion by regulating N-cadherin during neural crest migration

**DOI:** 10.1242/dev.147926

**Published:** 2017-07-01

**Authors:** Isabel Bahm, Elias H. Barriga, Antonina Frolov, Eric Theveneau, Paul Frankel, Roberto Mayor

**Affiliations:** 1 Department of Cell and Developmental Biology, University College London, London WC1E 6BT, UK; 2 London Centre for Nanotechnology, University College London, London WC1H 0AH, UK; 3 Centre for Cardiovascular Biology and Medicine, Division of Medicine, University College London, London WC1E 6JJ, UK

**Keywords:** PDGF, PDGFR, Neural Crest, EMT, Contact inhibition of locomotion, N-cadherin, Migration, *Xenopus*

## Abstract

A fundamental property of neural crest (NC) migration is contact inhibition of locomotion (CIL), a process by which cells change their direction of migration upon cell contact. CIL has been proven to be essential for NC migration in amphibians and zebrafish by controlling cell polarity in a cell contact-dependent manner. Cell contact during CIL requires the participation of the cell adhesion molecule N-cadherin, which starts to be expressed by NC cells as a consequence of the switch between E- and N-cadherins during epithelial-to-mesenchymal transition (EMT). However, the mechanism that controls the upregulation of N-cadherin remains unknown. Here, we show that platelet-derived growth factor receptor alpha (PDGFRα) and its ligand platelet-derived growth factor A (PDGF-A) are co-expressed in migrating cranial NC. Inhibition of PDGF-A/PDGFRα blocks NC migration by inhibiting N-cadherin and, consequently, impairing CIL. Moreover, we identify phosphatidylinositol-3-kinase (PI3K)/AKT as a downstream effector of the PDGFRα cellular response during CIL. Our results lead us to propose PDGF-A/PDGFRα signalling as a tissue-autonomous regulator of CIL by controlling N-cadherin upregulation during EMT. Finally, we show that once NC cells have undergone EMT, the same PDGF-A/PDGFRα works as an NC chemoattractant, guiding their directional migration.

## Abstract (precis)

**Summary:** PDGF-A and its receptor control *Xenopus* neural crest migration by promoting EMT and contact inhibition of locomotion, acting via N-cadherin regulation at early stages of development and working as chemoattractant later.

## INTRODUCTION

One of the most migratory cell types during early vertebrate development is cells of the neural crest (NC). The NC has a transient cell population, induced between the neural tube and surface ectoderm, which eventually gives rise to many different tissues, such as craniofacial cartilage and bone, cells of the peripheral nervous system, smooth muscle cells, tendons and pigment cells. Disruption of NC cell migration during development can lead to pathologies including craniofacial abnormalities, heart malformation and colonic aganglionosis (Hirschprung's disease), generally termed neurocristopathies (Feiner et al., 2001; Miyagawa-Tomita et al., 1991; Van de Putte et al., 2007).

A defining characteristic of NC cells is the epithelial-to-mesenchymal transition (EMT) they undergo to segregate from the neural tube and start their migration (Theveneau and Mayor, 2012). EMT is a cellular process that converts nonmotile epithelial cells to motile mesenchymal cells, defined by a change in cell-cell adhesion, polarity and the acquisition of migratory properties (Thiery et al., 2009). One of the migratory properties acquired during NC cell EMT is contact inhibition of locomotion (CIL) (Scarpa et al., 2015). This cellular process is characterized by a change in direction of migration upon cell-cell contact and is associated with embryonic processes, such as neuronal cell and macrophage dispersion, and collective migration of cranial NC cells (Carmona-Fontaine et al., 2008b; Davis et al., 2012; Kay et al., 2012; Stramer and Mayor, 2016). EMT in *Xenopus* and zebrafish cranial NC is defined by an acquisition of CIL, which has been linked to a switch from E- to N-cadherin (also known as cadherins 1 and 2, respectively) (Scarpa et al., 2015). This N-cadherin upregulation has been shown to be essential for CIL-dependent polarity in NC collective migration (Mayor and Etienne-Manneville, 2016; Theveneau et al., 2010, 2013). However, the mechanism of N-cadherin upregulation during NC migration remains unknown. The platelet-derived growth factor (PDGF) receptor tyrosine kinase pathway has been implicated in EMT during cancer invasion (Eckert et al., 2011; Jechlinger et al., 2006; Thiery and Sleeman, 2006), and it is essential for the correct development of several NC derivatives (Morrison-Graham et al., 1992; Soriano, 1997; Tallquist and Soriano, 2003). Furthermore, evidence suggests that the involvement of the PDGF pathway in the formation of NC derivatives is related to the control of NC cell migration and proliferation (Eberhart et al., 2008; He and Soriano, 2013; Smith and Tallquist, 2010). However, the specific mechanism by which PDGF controls the formation of NC-derived tissues has not been completely elucidated.

The PDGF signalling pathway is activated by five soluble, disulphide-linked, homo- or heteromeric ligands (PDGF-AA, PDGF-AB, PDGF-BB, PDGF-CC, PDGF-DD) that bind to three receptor tyrosine kinases (PDGFRα/Rα, PDGFRβ/Rβ, PDGFRα/Rβ), leading to the subsequent activation of downstream signalling cascades (Hoch and Soriano, 2003). These can affect a wide range of cellular events, such as proliferation, migration, survival and EMT. Functional *in vivo* interaction studies in mice demonstrated that platelet-derived growth factor A (PDGF-A) and PDGF-C activate platelet-derived growth factor receptor alpha (PDGFRα) signalling (Boström et al., 1996; Ding et al., 2004; Soriano, 1997). PDGFRα is expressed in cranial NC cells in *Xenopus*, zebrafish and mouse embryos (Ho et al., 1994; Liu et al., 2002b; Orr-Urtreger et al., 1992; Takakura et al., 1997; Fantauzzo and Soriano, 2016). PDGFRα signalling, together with its ligand PDGF-A, has been suggested to work as a chemotactic cue for NC cells (Eberhart et al., 2008; He and Soriano, 2013; Kawakami et al., 2011). Perturbations of PDGFRα signalling in mouse and zebrafish lead to severe defects in cranial NC cell-derived tissues, suggesting a role for PDGFRα signalling in the development of the NC towards its craniofacial targets (Eberhart et al., 2008; He and Soriano, 2013; Morrison-Graham et al., 1992; Soriano, 1997; Tallquist and Soriano, 2003). By contrast, PDGFRβ signalling does not seem to be required for NC cell development (Levéen et al., 1994; McCarthy et al., 2016; Tallquist et al., 2000). However, a recent publication showed that PDGFRα and PDGFRβ can form a functional heterodimer, and that double knockdown mutants exhibit a more severe craniofacial phenotype than those with either mutation alone (Fantauzzo and Soriano, 2016). Analysis of the downstream signalling binding sites of PDGFRα during mouse craniofacial development revealed the phosphatidylinositol-3-kinase (PI3K)/AKT signalling pathway as the primary signalling effector (Klinghoffer et al., 2001; McCarthy et al., 2013; Vasudevan et al., 2015). However, very little is known about early roles of PDGFRα signalling in cranial NC migration.

Here, we use *Xenopus* cranial NC cells to investigate the role of PDGF signalling in NC migration. We show that PDGF-A and its receptor PDGFRα are specifically co-expressed in pre-migratory and migratory NC cells. We find that PDGF-A works as a chemotactic signal for migratory, but not pre-migratory, NC cells. Analysis of this pre-migratory phenotype shows that inhibition of PDGF-A/PDGFRα blocks cell dispersion by downregulation of N-cadherin, which is required for CIL acquisition during EMT. Furthermore, we find that this novel role of PDGF signalling in the NC requires downstream activity of the PI3K/AKT signalling pathway.

## RESULTS

### PDGF-A and PDGFRα are co-expressed in the NC and are required for NC migration

**Fig. 1.**
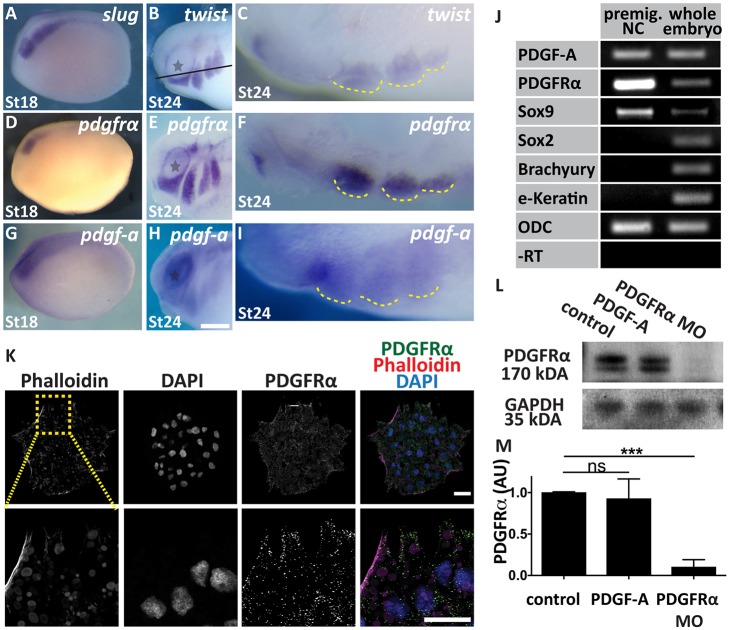
**NC cells express both PDGF-A and PDGFRα.** (A-I) Whole-mount *in situ* hybridization of *Xenopus* embryos. (A,D,G) Lateral view of stage 18 embryos showing expression of (A) *slug*, (D) *pdgfrα* and (G) *pdgf-a*. (B,E,H) Lateral view of stage 24 embryos showing migrating NC expressing (B) *twist*, (E), *pdgfrα* and (H) *pdgf-a*. Scale bar: 1 mm. Grey stars indicate the eyes. (C,F,I) Sections of embryos shown in B,E,H, respectively. The line in B indicates the level of the section. Yellow dashed lines outline cephalic NC streams. (J) RT-PCR analysis of *pdgf-a* and *pdgfrα* expression in NC dissected from stage 18 embryos (premig. NC) and whole embryos along with *Sox9* (NC marker), *Sox2* (neural plate marker), *brachyury* (mesoderm marker), *e-Keratin* (epidermis marker) and ODC (control, ornithine decarboxylase). (K) Immunostaining for PDGFRα (green), Phalloidin (red) and DAPI (blue) in NC explants. Scale bars: 20 µm. (L) Western blot analysis of PDGFRα in NC dissected from control embryos, embryos treated with PDGF-A or embryos injected with PDGFRα MO. GADPH was used as a loading control. (M) Band intensity normalized to the loading control. Data are mean±s.d. of three independent experiments. AU, arbitrary units. ns, not significant; ***P<0.001.

We first analysed the expression of PDGFRα and PDGF-A by *in situ* hybridization and RT-PCR. We found that PDGFRα is expressed in pre-migratory (stage 18) and migrating (stage 24) cranial NC cells, as shown by comparison with the specific NC markers *slug* and *twist* (Fig. 1A-F). Expression of *pdgf-a* was found in pre-migratory NC (Fig. 1G) and also in tissues surrounding the migrating NC (Fig. 1H,I), as previously described (Ho et al., 1994). To confirm this finding, we performed RT-PCR in NC dissected from stage 18 embryos (pre-migratory), and observed strong expression of *pdgfa* in the dissected tissue (Fig. 1J). To test for non-NC tissue contamination, we also performed RT-PCR for a neural plate marker (*Sox2*), an ectoderm marker (*e-Keratin*) and a mesoderm marker (*brachyury*), and for an NC marker (*Sox9*) as a positive control. We did not detect any of the non-NC tissue markers in our NC samples, which were positive for the NC marker (Fig. 1J). The expression of PDGFRα in the NC was further confirmed by immunostaining (Fig. 1K) and western blotting (Fig. 1L,M). These data strongly support the notion that PDGF-A and PDGFRα are co-expressed in the migrating NC.

**Fig. 2.**
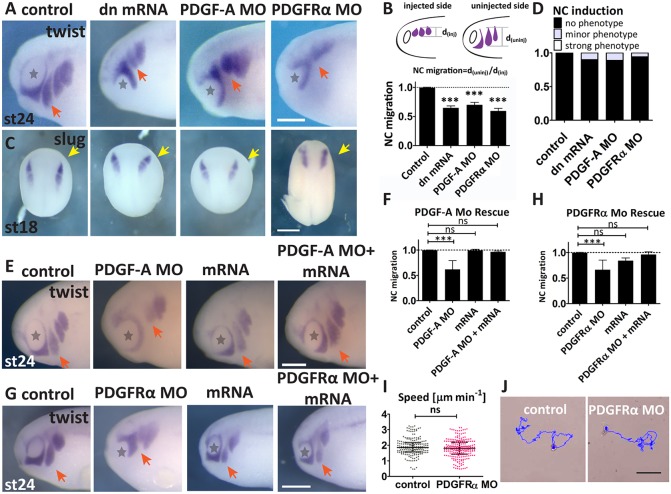
**PDGF-A and PDGFRα are required for NC migration *in vivo*.** (A-D) PDGF signalling depletion affects NC migration, but not specification. (A) *In situ* hybridization of *twist* in stage 24 embryos after the indicated treatments. Scale bars: 1 mm. (B) Diagram of quantification of NC migration; distance of the second (hyoid) NC stream on the injected side was normalized to that on the uninjected control side. Bar graph of NC migration in control (*n*=30), dominant-negative mRNA (dn mRNA) (PDGFRαw37, *n*=51), PDGF-A MO (*n*=47) and PDGFRα MO (*n*=28) embryos, from three independent experiments. (C) *In situ* hybridization of *slug* in stage 18 embryos after the indicated treatments. Dorsal view, yellow arrows indicate the site of injection. Scale bar: 500 μm. (D) Analysis of NC induction as shown in C. (E) *In situ* hybridization of *twist* in stage 24 embryos after the indicated treatments. Scale bar: 1 mm. (F) PDGF-A MO rescue experiment showing specificity of MO treatment. NC migration in control (*n*=30), PDGF-A MO (*n*=24), mouse PDGF-A mRNA (*n*=28), PDGF-A MO and mouse PDGF-A mRNA (*n*=27) embryos. (G) *In situ* hybridization of *twist* in stage 24 embryos after the indicated treatments. Scale bar: 1 mm. Stars indicate the eye and red arrows indicate the neural crest streams. (H) PDGRFα MO rescue, showing the specificity of the MO treatment. NC migration in control (*n*=20), PDGFRα MO (*n*=36), mouse PDGFRα mRNA (*n*=22), and PDGFRα MO+mouse PDGFRα mRNA (*n*=41) embryos. (I) Analysis of NC motility (µm/sec) in control (*n*=202) and PDGFRα MO-injected (*n*=208) single cells from three independent experiments. Scatter plots show median and interquartile range. (J) Images showing representative tracks of nuclear RFP-injected cells over 5 h. Scale bar: 50 µm. In all bar graphs, data are mean±s.e.m. ns, not significant; ***P<0.001.

In order to analyse the role of PDGF-A/PDGFRα in NC migration, we developed an anti-sense morpholino (PDGFRα MO), which reduced PDGFRα protein levels with high efficiency (Fig. 1L,M). In addition, we used previously published tools, such as a morpholino against the receptor ligand (PDGF-A MO) (Nagel et al., 2004) and a dominant-negative form of PDGFRα (PDGFRαw37 mRNA) (Ataliotis et al., 1995). Depletion of PDGFRα, or its ligand PDGF-A, led to the significant inhibition of NC cell migration *in vivo* (Fig. 2A,B), without affecting NC specification (Fig. 2C,D), suggesting that it affected a specific mechanism during migration without any effect on NC cell induction. To verify the specificities of the receptor and ligand morpholinos, we co-injected them with mouse mRNA, which does not hybridize with the *Xenopus laevis* target sequence in the morpholinos (see Materials and Methods), and analysed the effect on NC migration. For both morpholinos (PDGF-A MO and PDGFRα MO), co-injection with their respective mRNAs rescued NC migration back to wild-type levels (Fig. 2E-H).

To investigate possible changes in NC cell motility resulting from PDGF signalling depletion, NC cells were dissociated and single-cell migration was monitored using time-lapse microscopy. Analysis of cell motility did not reveal any difference in cell velocity between PDGFRα MO-injected and control cells (Fig. 2I,J), suggesting that inhibition of PDGFRα does not affect the motility of single cells. Taken together, these data indicate that inhibition of PDGF-A/PDGFRα signalling impairs NC migration *in vivo* and that this phenotype is not caused by an effect on cell motility per se.

### Migratory but not pre-migratory NC cells chemotax towards PDGF-A

**Fig. 3.**
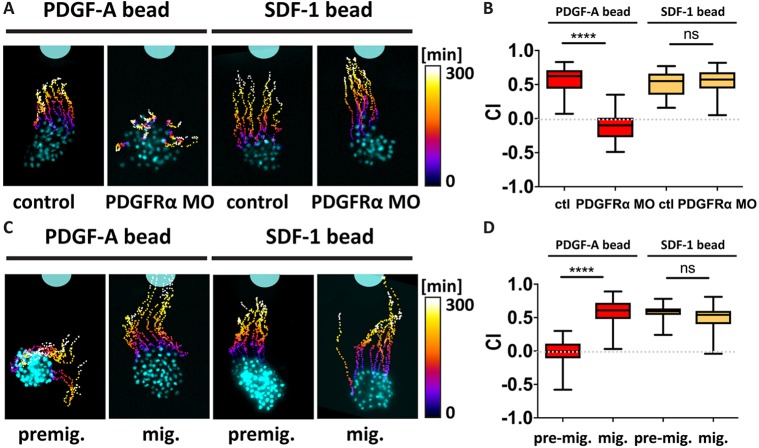
**Only migratory NC cells migrate towards PDGF-A.** (A) *In vitro* chemotaxis assay towards PDGF-A and SDF-1 protein-coated beads; representative images of nuclear fluorescence-labelled NC clusters (cyan) with time-coded tracks from t=0 min to t=300 min are shown. (B) Chemotaxis index (CI) of migratory NC with PDGF-A protein [control (*n*=50), PDGFRα MO (*n*=50)] and SDF-1 protein [control (*n*=47), PDGFRα MO (*n*=50)] beads. (C) *In vitro* chemotaxis assay of pre-migratory (premig., stage 18) and migratory (mig., stage 22) NC clusters towards PDGF-A protein-coated beads; representative images of nuclear fluorescence-labelled NC clusters (cyan) with time-coded tracks from t=0 min to t=300 min are shown. (D) CI of migratory NC with PDGF-A protein [pre-mig. (*n*=131), mig. (*n*=115)] and SDF-1 protein [pre-mig. (*n*=100), mig. (*n*=100)] beads. Box plots show the median and 25th and 75th percentiles, whiskers are the minimum and maximum values. ns, not significant; ****P<0.0001.

Inhibition of migration by depletion of PDGF signalling could be due to decreased chemotaxis, as PDGF-A has been suggested to work as a chemoattractant for NC cells in zebrafish and mouse (Eberhart et al., 2008; Kawakami et al., 2011). To test whether impaired NC migration after PDGFRα inhibition is caused by inhibition of chemotaxis towards PDGF-A, we used a previously published *in vitro* chemotaxis bead assay (Theveneau and Mayor, 2011; Theveneau et al., 2010). Control and PDGFRα MO-injected explants were plated in close proximity to PDGF-A protein-coated beads, and their migratory behaviour was analysed by time-lapse microscopy. Indeed, control explants showed strong chemotaxis towards the PDGF-A source (Fig. 3A,B; Movie 1). Furthermore, depletion of PDGFRα inhibited chemotaxis towards the PDGF-A beads (Fig. 3A,B; Movie 1), indicating that PDGF-A might work as a chemoattractant. To test whether the inhibition of chemotaxis in PDGFRα MO-injected NC is caused by loss of transmission of the chemotaxis signal and not by loss of migratory behaviour, we investigated whether PDGFRα-depleted explants are still able to migrate towards the known NC chemoattractant stromal cell-derived factor 1 (SDF-1) (Belmadani, 2005; Olesnicky Killian et al., 2009; Theveneau et al., 2010). We observed no difference in migration towards the SDF-1 source between the PDGFRα-depleted and control NC explants (Fig. 3A,B; Movie 1), indicating that the effect of PDGF-A/PDGFRα chemotaxis is independent of the role of SDF-1 in NC cell migration. Taken together, these results suggest that PDGF-A might work as a chemoattractant in *Xenopus* NC cells.

As PDGFRα is expressed at pre-migratory and migratory stages, we performed a temporal analysis of the chemotaxis response. We found that although migratory NC cells (stage 22) exhibited a strong chemotaxis response (Fig. 3C, mig.), pre-migratory NC cells (stage 18) did not migrate towards PDGF-A (Fig. 3C, premig.; Movie 2). To verify that this lack of chemotaxis in pre-migratory NC cells is not due to a general inability of pre-migratory NC cells to react to a chemotactic cue, we performed a pre-migratory chemotaxis assay with SDF-1. No change in chemotaxis behaviour towards the SDF-1 protein source in either pre-migratory or migratory NC explants was observed (Fig. 3C,D; Movie 2). Taken together, these data indicate that chemotaxis of NC towards PDGF-A is present only in migrating NC cells, and its absence in pre-migratory cells suggests a role of PDGF-A/PDGFRα at these early stages that does not involve chemotaxis. As a role of PDGF on NC chemotaxis has already been described (Eberhart et al., 2008; He and Soriano, 2013; Kawakami et al., 2011; Shellard and Mayor, 2016), we decided to focus our investigation on the early nonchemotactic role of PDGF.

### PDGF-A/PDGFRα controls dispersion via N-cadherin regulation

**Fig. 4.**
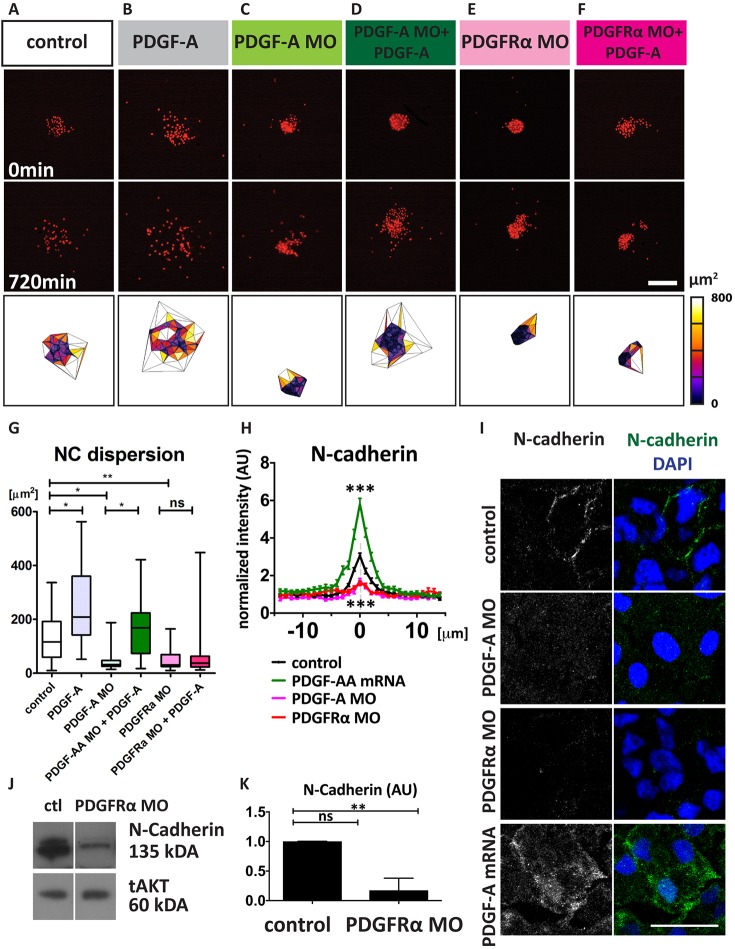
**PDGF signalling affects NC dispersion and N-cadherin levels.** (A-F) Nuclear fluorescence (nuclear RFP) of NC explants cultured *in vitro* for the indicated times with the indicated treatments. Scale bar: 100 µm. Bottom row shows analysis of cell dispersion using Delaunay triangulation at 720 min. (G) NC dispersion analysis based on average Delaunay triangulation area (μm^2^) of control (*n*=43), PDGF-A protein (50 ng/ml, *n*=31), PDGF-A MO (*n*=18), PDGF-A MO+PDGF-A protein (*n*=13), PDGFRα MO (*n*=30), PDGFRα MO+PDGF-A protein (*n*=29) explant clusters from at least three independent experiments. Box plots show the median and 25th and 75th percentiles, whiskers are the minimum and maximum values. (H) Analysis of immunostaining against N-cadherin; pixel intensity across cell-cell contacts (0 μm), normalized to average cell background level in control (*n*=43), PDGF-A MO (*n*=50), PDGFRα MO (*n*=61), PDGF-A mRNA (*n*=50) cells. Data are mean±s.e.m. AU, arbitrary units. (I) Representative projections of confocal images of immunostaining against N-cadherin (green) and DAPI (blue) in control, PDGF-A MO, PDGFRα MO and PDGF-A mRNA-injected NC cells. Scale bar: 20 μm. (J) Western blot against N-cadherin from NC explants taken from control embryos or embryos injected with PDGFRα MO. tAKT was used as a loading control. (K) Normalized levels of N-cadherin protein levels. Data are mean±s.d. from three independent experiments. ns, not significant;*P<0.05 **P<0.01; ***P<0.001.

Initial migration of NC requires EMT; therefore, we tested the possibility that impaired NC migration could be caused by defects in EMT. To assess the potential influence of PDGF signalling on EMT, NC cell dispersion was analysed in the pre-migratory NC (stage 18). Nuclear fluorescence-labelled NC cell clusters were monitored by time-lapse microscopy, and cell dispersion was quantified by measuring the distance between the nucleus of an NC cell and that of its nearest neighbour using Delaunay triangulation (Carmona-Fontaine et al., 2011). Inhibition of PDGF-A (Fig. 4C,G) and PDGFRα (Fig. 4E,G) drastically reduced NC cell dispersion compared with control explants (Fig. 4A,G), confirming our previous observation that both PDGFRα and PDGF-A are co-expressed by NC cells and are functionally active in these cells (Movie 3). Moreover, addition of the ligand, PDGF-A, further increased cell dispersion of wild-type NC cells (Fig. 4B,G; Movie 3) or cells depleted of PDGFA (Fig. 4D), but it was unable to promote dispersion in cells lacking PDGFRα (Fig. 4F,G; Movie 3), showing again the specificity of the inhibition by PDGF-A MO and PDGFRα MO. This suggests that PDGF-A/PDGFRα signalling regulates NC dispersion at early pre-migratory stages.

One of the outcomes of EMT is that it promotes cell dispersion by reducing cell-cell adhesion or increasing cell motility. As we showed that depletion of PDGF-A/PDGFRα inhibits cell dispersion without affecting cell motility, we decided to analyse cell-cell adhesion. Members of the cadherin protein family are key cell-cell adhesion molecules, and a switch from E- to N-cadherin is essential for NC migration (Scarpa et al., 2015; Rogers et al., 2013). Therefore, we investigated the impact of PDGFRα signalling on N- and E-cadherin levels by western blot analysis of pre-migratory cells. We observed a reduction of N-cadherin protein levels in PDGFRα MO-injected NC cells (Fig. 4J,K), but no change in E-cadherin (Fig. S1A,B). The specific decrease in N-cadherin, but not E-cadherin, was further confirmed by immunofluorescence in PDGFRα-depleted NC cells (Fig. 4H,I; Fig. S1C,D). Additionally, we observed a similar decrease in N-cadherin staining in PDGF-A-depleted cells (Fig. 4H,I, PDGF-A MO) and an opposite increase after PDGF-A mRNA injection (Fig. 4H,I). These data suggest that PDGF-A/PDGFRα signalling controls NC EMT and cell dispersion at pre-migratory stages by regulating N-cadherin levels.

### N-cadherin-dependent CIL is regulated by PDGF-A/PDGFRα signalling

**Fig. 5.**
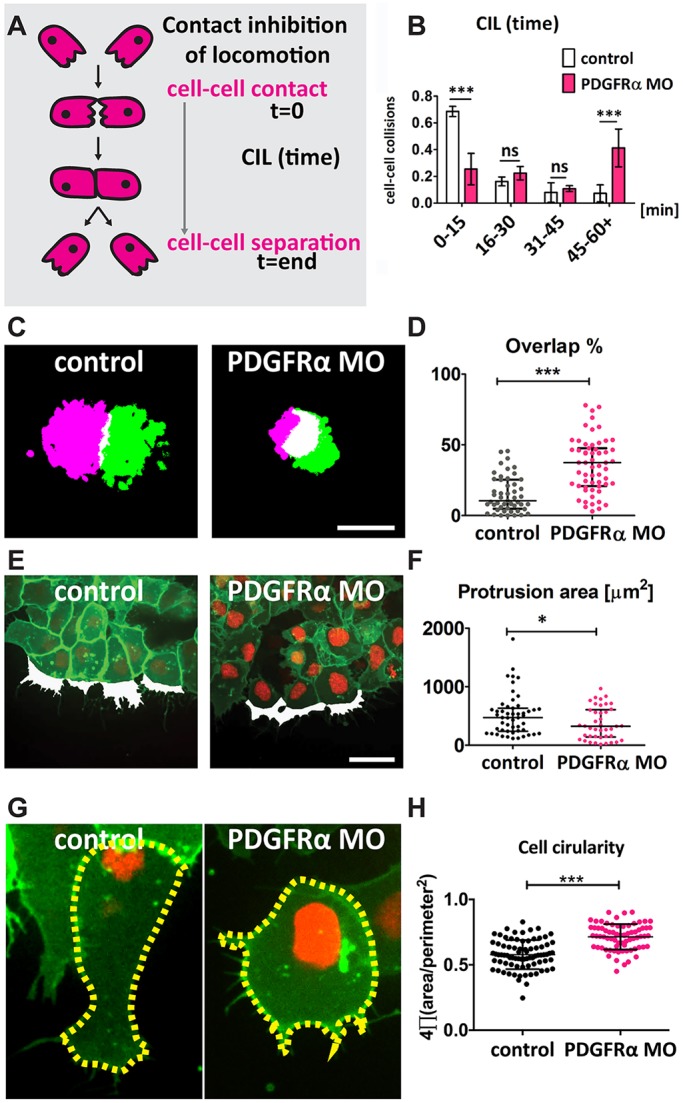
**PDGF signalling controls CIL.** (A,B) Single cell collision assay of the time between first contact and separation. (A) Representative diagram of CIL. (B) PDGFRα MO explants have impaired CIL, as indicated by the duration of control-control (*n*=58) and PDGFRα MO-PDGFRα MO CIL events (*n*=63); data are mean±s.d. from three independent experiments. (C) CIL assay, in which the overlap between two differentially labeled explants is analyzed. Thresholded images of explant invasion assay. Scale bar: 100 µm. (D) Overlap percentage between two NC explants of control (*n*=52) and PDGFRα MO (*n*=59) explants from three independent experiments. Scatter plots show median and interquartile range. (E) Protrusions formed away from the cell contact (labelled in white). Scale bar: 30 μm. (F) Protrusion area (μm^2^) of control (*n*=51) and PDGFRα MO (*n*=41) cells. Scatter plots show median and interquartile range. (G) Circularity index; representative examples of circularity (indicated by yellow dashed line) of control and PDGFRα MO-injected NC cells. (H) Circularity of control (*n*=79) and PDGFRα MO (*n*=73)-injected NC cells. ns, not significant; *P<0.05; ***P<0.001.

Our results show that inhibition of PDGF-A/PDGFRα reduces the levels of N-cadherin protein at cell-cell contacts and, at the same time, reduces cell dispersion. How can this decrease in N-cadherin be related to a reduction in cell dispersion? It is known that blocking N-cadherin leads to a loss of CIL behaviour in NC (Theveneau et al., 2010). Hence, we hypothesized that decreased dispersion in PDGFRα MO-injected explants could be caused by N-cadherin-dependent loss of CIL. To address this, we used three different assays to analyse CIL (Carmona-Fontaine et al., 2008b; Moore et al., 2013; Scarpa et al., 2015). All of these assays were performed using pre-migratory NC cells (stage 18). First, when two cells undergoing CIL collide they remain briefly in contact and then they move away from each other (Fig. 5A); however, if CIL is impaired the two colliding cells remain in contact for longer (Stramer and Mayor, 2016). We measured the time that pairs of colliding cells remain together as an outcome of CIL. Our results show that cells injected with PDGFRα MO remain in contact significantly longer compared with control cells (Fig. 5B), with some PDGFRα MO-injected cells never separating even after 10 h of culture, indicating an impairment in the CIL response. Second, when two cell explants that exhibit CIL are confronted they do not overlap; therefore, an overlap between adjacent explants is an indication of CIL impairment (Stramer and Mayor, 2016). Two NC explants fluorescently labelled with distinct colours, Fluorescein-Dextran and Rhodamine-Dextran, were cultured at a short distance and the overlapping area between them was analysed. Although control explants did not overlap, a clear overlap was observed in explants in which the PDGFRα was inhibited, indicating a clear reduction in CIL response (Fig. 5C,D). Third, a direct consequence of CIL is the acquisition of cell polarity, where cells extend a larger protrusion away from the contact and become elongated, which has been linked to N-cadherin-dependent cell adhesion (Scarpa et al., 2015; Theveneau et al., 2010). To assess whether inhibition of PDGFRα changes polarity, we measured the protrusion area away from the cell contact and cell circularity. We found a significantly lower protrusion area and higher circularity in PDGFRα MO-injected cells compared with control cells (Fig. 5E-H), suggesting a change in polarity. Overall, these data support the idea that PDGFRα is controlling CIL via N-cadherin regulation.

### PDGF-A/PDGFRα controls NC migration via the PI3K/AKT signalling pathway

It is known that PDGFR can activate several signalling pathways, such as the PI3K, MAPK, PKC, JAK-STAT and Src pathways (Demoulin and Essaghir, 2014). Therefore, we were interested in identifying the pathways involved in controlling CIL/N-cadherin in pre-migratory NC cells. Studies in mouse and zebrafish during craniofacial NC migration suggested PI3K signalling as the main downstream effector of PDGF signalling (He and Soriano, 2013; Klinghoffer et al., 2001; Vasudevan et al., 2015). Therefore, we asked whether the same pathways were activated in pre-migratory NC cells during CIL.

**Fig. 6.**
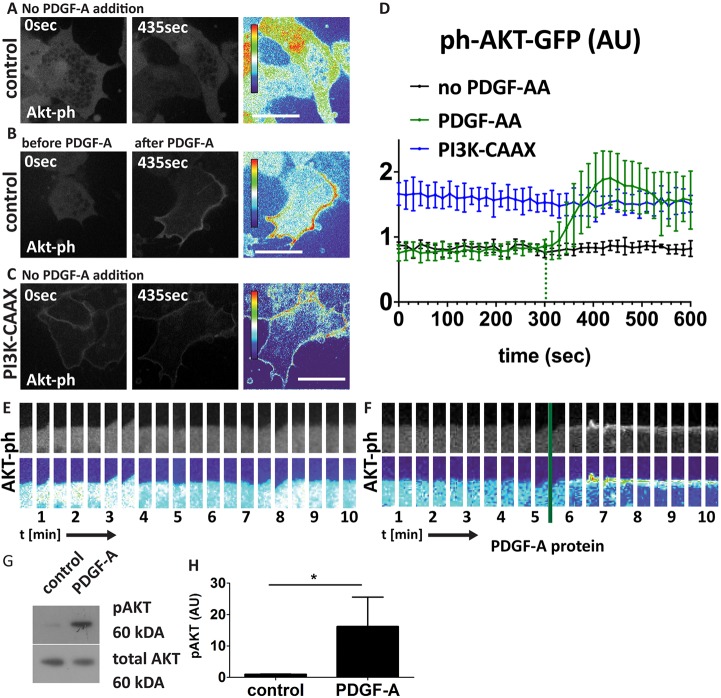
**AKT signalling downstream of PDGFRα.** (A-C) Confocal images of ph-AKT-GFP (Akt-ph) mRNA-injected NC cells at t=0 s and t=425 s. Scale bars: 20 μm. (A,E) Control, no addition of PDGF-A protein. (B,F) PDGF-A protein (50 ng/ml) added at 300 s. (C) PI3K-CAAX mRNA-injected NC with no addition of PDGF-A protein. (D) Analysis of ph-AKT-GFP pixel intensity at the membrane normalized to cytoplasmic ph-AKT-GFP levels over time. PDGF-A addition control (PDGF-AA, *n*=12), no PDGF-A addition control (no PDGF-AA, *n*=7), PI3K-CAAX (*n*=10); data are mean±s.d. from three independent experiments. (E,F) Time course (10 min) of images at the cell membrane showing membrane localization of ph-AKT-GFP (E) with no PDGF-A addition and (F) after PDGF-A addition. (G) Western blot analysis of pAKT (Ser437) in control and PDGF-A protein-treated (50 ng/ml for 60 min) NC explant lysates. tAKT was used as a loading control. (I) Band intensity normalized to loading control. Data are mean±s.d. from three independent experiments. AU, arbitrary units. ns, not significant; *P<0.05.

To investigate the role of PI3K as a downstream component of PDGFRα signalling, we expressed a biosensor (ph-AKT-GFP) of PI3K activity, consisting of AKT pleckstrin homology (ph) domain fused to GFP (Montero et al., 2003), in NC cells and analysed PI3K/AKT activity by high time resolution microscopy. Activation of PI3K results in the addition of a phosphate molecule to phosphoinositides, generating phosphatidylinositol 3,4,5-trisphosphate (PIP3). ph-AKT-GFP has a high and specific affinity for PIP3 and therefore translocates to the plasma membrane upon binding PIP3 (Hoch and Soriano, 2003). Thus, a change in GFP intensity from the cytosolic to the membrane-bound form can be used as a read-out of PI3K pathway activation. The treatment of ph-AKT-GFP-expressing cells with PDGF-A protein induced a clear increase in membrane GFP intensity compared with control cells not treated with PDGF-A protein (Fig. 6A,B,D-F; Movie 4). As a positive control, we co-injected the cells with PI3K-CAAX mRNA, a dominant active form of PI3K, which resulted in a strong membrane GFP response without the addition of PDGF-A protein (Fig. 6C,D).

To further examine the role of PI3K/AKT signalling, we tested for endogenous pathway activation by western blot analysis of phosphorylated AKT (pAKT). Treatment with PDGF-A protein led to an increase in AKT phosphorylation in NC cells (Fig. 6G,H), suggesting an involvement of PI3K/AKT signalling downstream of PDGF-A/PDGFRα. We observed no decrease in AKT phosphorylation in PDGFRα MO-injected cells, probably because the basal levels of AKT are already too low in control cells.

**Fig. 7.**
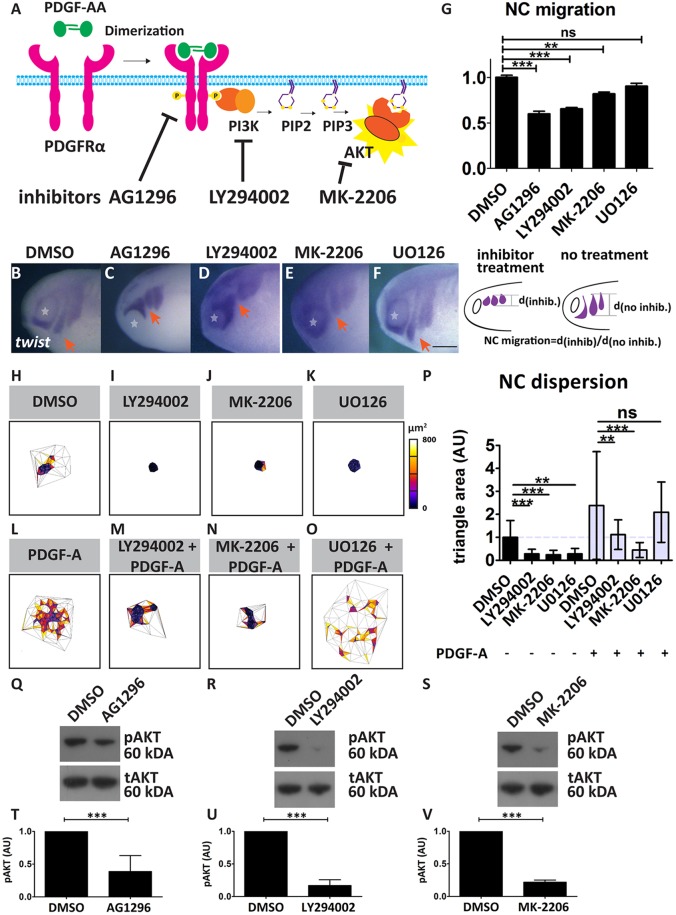
**Small molecule inhibition of PI3K/AKT signalling.** (A) Schematic of the PDGF-PI3K-AKT signalling axis. Signalling is activated upon PDGF ligand (PDGF-AA) binding to the PDGF receptor (PDGFRα), inducing receptor dimerization and subsequent autophosphorylation. PI3K is activated, leading to the phosphorylation of phosphoinositol (PI) residues in the plasma membrane, converting PIP2 to PIP3. Downstream effector kinase AKT binds [with its pleckstrin homology (ph) domain] to PIP3 residues and is activated. (B-F) *In situ* hybridization for *twist* in stage 24 embryos treated with the indicated inhibitors from stage 14: (B) DMSO control, (C) AG1296 (20 μM), (D) LY294002 (40 μM), (E) MK-2206 (100 μM), (F) UO126 (100 μM). Scale bar: 100 μm. Stars indicate the eye and arrows indicate the neural crest stream. (G) NC migration normalized to the control average of each experiment in DMSO control (*n*=256), AG1296 (*n*=101), LY294002 (*n*=40), MK-2206 (*n*=106) and UO126 (*n*=68) embryos. Data are mean±s.e.m. from three independent experiments. Scheme showing how the migration was quantified is shown underneath the graph. (H-O) Dispersion analysis, using Delaunay triangulation, of NC cultured *in vitro* for 720 min with the indicated treatments. (P) Analysis of NC dispersion, showing average Delaunay triangulation area normalized to the control of each experiment. Control (*n*=92), LY294002 (5 μM, *n*=21), MK-2206 (5 μM, *n*=16), UO126 (25 μM, *n*=14), control and PDGF-A (*n*=55), LY294002 (5 μM) and PDGF-A (*n*=25), MK-2206 (5 μM) and PDGF-A (*n*=35), UO126 (25 μM) and PDGF-A (*n*=19) explants; for all PDGF-A conditions, a PDGF-A concentration of 50 ng/ml was used. Data are mean±s.d. (Q-S) Western blots against pAKT using lysates of whole embryos treated with small molecule inhibitors as indicated. tAKT was used as a loading control. (T-V) Quantification of the western blots shown in Q-S, respectively; intensity of pAKT normalized to tAKT control. Data are mean±s.d. of three independent experiments. AU, arbitrary units. ns, not significant; **P<0.01; ***P<0.001.

To investigate the role and specificity of PI3K/AKT signalling in NC migration, we used pharmacological inhibitors (Fig. 7A) of PDGFR (AG1296), PI3K (LY294002), AKT (MK2206) and MEK (UO126). *In vivo* treatment with PDGFR, PI3K and AKT inhibitors at the pre-migratory stage led to inhibition of NC migration (Fig. 7B-G), supporting our earlier findings. No significant effect of MEK inhibition on NC migration was detected (Fig. 7F,G). Next, we investigated the effect of PI3K/AKT on pre-migratory NC cell dispersion *in vitro*, which is considered as a CIL assay (Stramer and Mayor, 2016). Treatment with PI3K (Fig. 7I,P, black bars) and AKT (Fig. 7J,P, black bars) inhibitors led to inhibition of NC cell dispersion compared with control NC cells (Fig. 7H,P, black bars). Remarkably, treatment with the MEK inhibitor also resulted in a reduction in NC cell dispersion (Fig. 7K,P, black bars), but had no effect on NC cell migration *in vivo* (Fig. 7F,G). As some of these inhibitors could also have nonspecific or off-target effects on other pathways present in the NC, different to the one activated by PDGF-A, we proceeded to use the inhibitors after treating the cell clusters with PDGF-A protein. As previously shown, we observed that treatment of NC cells with PDGF-A led to cell dispersion (Fig. 7L,P, grey bars). However, this dispersion was dramatically impaired when PDGF-A-treated cells were co-incubated with inhibitors of PI3K (Fig. 7M,P, grey bars) and AKT (Fig. 7N,P, grey bars), but not with the MEK inhibitor (Fig. 7O,P, grey bars). These results show that PDGF-A promotes cell dispersion in a PI3K/AKT-dependent manner, but independently of MEK, which is consistent with the effect of the inhibitors of these pathways on NC migration *in vivo*. The results using UO126, a MEK inhibitor, suggest some off-target effects *in vitro*, but not *in vivo*, possibly because higher levels of the inhibitor are reached in cells directly exposed to the culture medium *in vitro* but not *in vivo*. Immunoblotting for pAKT confirmed a decrease after PDGFR inhibitor treatment (Fig. 7Q,T), similar to treatment with the inhibitors of PI3K (Fig. 7R,U) and AKT (Fig. 7S,V). Taken together, these data show that PDGF-A/PDGFRα controls NC migration and dispersion via the PI3K/AKT signalling pathway.

### N-cadherin-dependent CIL is regulated via PI3K/AKT signalling downstream of PDGF-A/PDGFRα signalling

**Fig. 8.**
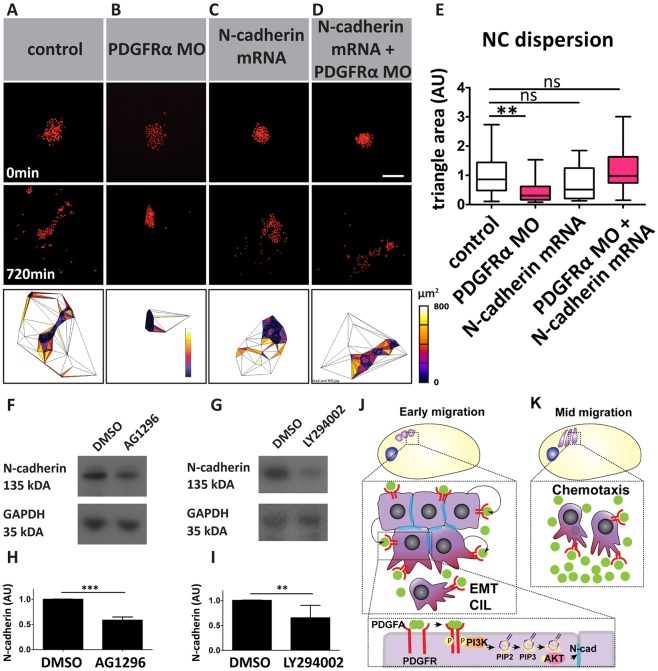
**PDGFRα controls dispersion via PI3K/AKT in an N-cadherin-dependent manner.** (A-D) Nuclear fluorescence (nuclear RFP) of NC explants cultured *in vitro* for the indicated time with the indicated treatments. Scale bar: 100 µm. Bottom row shows analysis of cell dispersion using Delaunay triangulation at 720 min. (E) NC dispersion analysis based on average Delaunay triangulation area (μm^2^) of control (*n*=41), PDGFRα MO (*n*=19), N-cadherin-GFP mRNA (*n*=23) and N-cadherin-GFP mRNA+PDGFRα MO (*n*=23) explants. Box plots show median and 25th and 75th percentiles, whiskers are the minimum and maximum values. (F,G) Western blot against N-cadherin using lysates of NC cells treated with DMSO, AG1296 (20 μM, *n*=3 blots) or LY294002 (40 μM, *n*=3 blots). GADPH was used as a loading control. (H,I) Band intensity of N-cadherin normalized to the loading control. Data are mean±s.d. of three independent experiments. AU, arbitrary units. ns*,* not significant; **P<0.01; ***P<0.001. (J,K) Schematic of the effects of PDGFRα/PDGF-A on (J) early and (K) mid NC migration. See text for further details.

It is well-established that a cell cluster undergoing CIL will disperse (Davis et al., 2015; Scarpa et al., 2015; Stramer and Mayor, 2016; Villar-Cerviño et al., 2013); conversely, the inhibition of CIL causes impaired dispersion, as observed in PDGF-A/PDGFRα MO-injected NC explants (Fig. 4C-G; Movie 3). To assess whether this impaired dispersion is caused by a lack of N-cadherin, we co-injected PDGFRα MO with N-cadherin mRNA. Interestingly, co-injection of PDGFRα MO and N-cadherin mRNA was sufficient to rescue the inhibition of dispersion induced by PDGFRα MO (Fig. 8A-E; Movie 5). This observation indicates that PDGF-A/PDGFRα signalling controls N-cadherin-dependent CIL in NC cells.

Finally, to investigate whether the N-cadherin regulation that is controlled by PDGF-A/PDGFRα (Fig. 4H-K) is also PDGFR-PI3K/AKT dependent, we treated embryos with a PDGFR or PI3K inhibitor, and analysed N-cadherin levels by western blotting at pre-migratory stages. Both inhibitor treatments reduced N-cadherin protein levels (Fig. 8F-I). In conclusion, these results demonstrate that the tissue-intrinsic PDGF-A/PDGFRα-PI3K-AKT signalling controls N-cadherin levels, which, in turn, are required for CIL during NC EMT.

## DISCUSSION

### PDGF-A and PDGFα are functionally linked in the NC

Our results point to a double role of PDGF-A/PDGFRα in *Xenopus* NC development. At early migratory stages (Fig. 8J), NC cells express PDGFRα and its ligand PDGF-A, which activates the PI3K/AKT pathway in a tissue-autonomous manner, leading to upregulation of N-cadherin at the cell contact; this increase in N-cadherin is sufficient to promote CIL and cell dispersion during EMT (Theveneau et al., 2010; Scarpa et al., 2015). Once EMT has started and the NC cells are migrating (Fig. 8K), the NC senses PDGF-A from the surrounding tissues, which induces chemotaxis and promotes directional NC migration as described previously in other animal models (Eberhart et al., 2008; He and Soriano, 2013; Kawakami et al., 2011).

Consistent with our data, expression of PDGFRα has been reported to be NC cell specific in mouse, zebrafish and *Xenopus* (Ho et al., 1994; Liu et al., 2002b; Orr-Urtreger and Lonai, 1992). However, in contrast to our finding that PDGF-A is expressed in *Xenopus* cranial NC, the expression of PDGF-A in other animal models has so far been attributed only to the NC cells surrounding tissues (Ho et al., 1994; Liu et al., 2002a; Orr-Urtreger and Lonai, 1992). In support of PDGF-A being produced by the NC, we show that depletion of PDGF-A in the NC by a morpholino inhibited NC dispersion *in vitro*, in a condition in which the only possible source of PDGF is the NC cells. So far, analysis of PDGF-A expression in *Xenopus* migratory NC has only been performed using radioactive *in situ* hybridization, in which expression can be confused with the background signal (Ho et al., 1994). We confirmed our results on the co-expression of PDGFRα and PDGF-A by using RT-PCR, which is a much more sensitive technique than *in situ* hybridization. Our data suggest that revisiting studies on PDGF-A expression during early cephalic NC migration in other model organisms with more recent and sensitive approaches would be worthwhile.

Various interactions between different ligands (PDGF-A, PDGF-AB, PDGF-BB, PDGF-CC, PDGF-DD) and receptors (PDGFRα/Rα, PDGFRβ/Rβ, PDGFRα/Rβ) have been described *in vitro*, but only the depletion of PDGF-A and PDGF-C, upstream of PDGFRα signalling, have been shown to be functionally important during mouse embryonic development (Boström et al., 1996; Ding et al., 2004; Soriano, 1997). We cannot rule out a potential role of PDGF-C in *Xenopus* NC migration; nonetheless, a PDGF-C ligand has so far not been described for *Xenopus laevis*. Although PDGFRβ expression has yet to be determined during *Xenopus laevis* development, PDGFRα and PDGFRβ can form a heterodimer that has been shown to be functionally active during NC migration (Fantauzzo and Soriano, 2016; Klinghoffer et al., 2002; Richarte et al., 2007), and future investigations should look at the potential role of PDGFRβ during NC migration in the *Xenopus*.

We showed that loss of function of both PDGF-A and PDGFRα inhibits NC cell migration *in vivo* using morpholinos against PDGF-A and PDGFRα, a dominant-negative form of PDGFRα and pharmacological inhibition of PDGFR phosphorylation with AG1296. The morpholinos and dominant-negative form of PDGFRα did not affect NC specification, excluding that the inhibition of NC migration is caused by a defect in NC specification. In line with our data, mouse and zebrafish PDGFRα knockdown studies have shown defects in cranial and cardiac NC-derived tissues (Eberhart et al., 2008; Soriano, 1997; Tallquist and Soriano, 2003). In zebrafish and mouse cranial NC, PDGF-A has been suggested as a chemokine during migration (Eberhart et al., 2008; Kawakami et al., 2011). Here, we show that PDGF-A can work as a chemoattractant, at least *in vitro*, for migratory, but not pre-migratory, NC. Why pre-migratory NC cannot undergo chemotaxis towards PDGF remains to be investigated.

### PDGF signalling and CIL

Analysis of the cellular behaviour controlled by PDGFRα revealed that NC dispersion is inhibited by PDGFRα depletion in pre-migratory stages. Consistent with our observation, conditional PDGFRα knockout mice have shown defects in explant outgrowth (He and Soriano, 2013). NC cells are known to undergo EMT-like dispersion *in vitro* (Kuriyama et al., 2014) due to a switch in the cell-cell adhesion molecules E-cadherin to N-cadherin (Scarpa et al., 2015). In line with these data, we demonstrate that PDGFRα signalling controls NC cell-cell adhesion by regulating N-cadherin levels. Importantly, overexpression of N-cadherin mRNA resulted in rescue of inhibition of dispersion, strongly suggesting that PDGFRα signalling works upstream of N-cadherin levels.

N-cadherin has been shown to be required for CIL, and a cell-cell adhesion complex formed by N-cadherin, p120, α-catenin and β-catenin is transiently assembled upon cell-cell interactions in cranial NC (Kuriyama et al., 2014; Theveneau et al., 2010). More recently, the acquisition of CIL behaviour has been linked to EMT and a switch to N-cadherin in *Xenopus* cranial NC cell migration (Scarpa et al., 2015). In context with these findings, we demonstrate that PDGFRα MO-injected NC cells were not able to undergo efficient CIL (Fig. 5), supporting the hypothesis that PDGFRα is regulating CIL. N-cadherin expression promotes polarization of RAC1 activity towards the leading edge during CIL, and N-cadherin-depleted cells display a reduction in protrusion size (Theveneau et al., 2010). As expected, owing to the reduction in N-cadherin, PDGFRα MO-injected NC cell explants displayed a decrease in protrusion area, indicating a loss of polarity. This suggests that PDGFRα signalling controls EMT in a CIL-dependent manner by regulating N-cadherin levels. NC cell EMT and migration does have many similar characteristic with malignant cancer invasion. In line with this and our data, PDGF signalling has been implicated in EMT during cancer invasion (Eckert et al., 2011; Jechlinger et al., 2006; Thiery and Sleeman, 2006). Furthermore, mouse studies reported that conditional *pdgfra* knockout in NC, using a Wnt1-Cre1 driver, resulted in platogenesis defects, linked to delayed migration of NC in the frontonasal prominence (He and Soriano, 2013; Tallquist and Soriano, 2003). This delayed migration in mouse could be consistent with the observed phenotype of CIL reduction in PDGFRα-depleted NC.

### Signalling downstream of PDGF in NC

Using a biosensor and pharmacological inhibition, we were able to link PDGFRα signalling to the PI3K/AKT downstream pathway. PI3K signalling downstream of PDGFRα appears to be a conserved mechanism in development. In mouse, PDGFRα depletion of PI3K activation results in abnormalities in craniofacial development (Klinghoffer et al., 2002). Also, an increase in PI3K signalling is able to rescue craniofacial development in zebrafish from a PDGFRα knockdown background (McCarthy et al., 2013). Although these studies focus on the later, frontonasal migration of cranial NC cells, PI3K/AKT cytoplasmic signalling appears to be conserved for NC development.

Spatial PI3K activation in the leading edge of migrating cells has been shown to be a crucial intracellular guidance cue in cell culture assays and *Dictyostelium* (Cain and Ridley, 2009; Merlot and Firtel, 2003; Yamaguchi et al., 2015). Contrary to this, we did not detect high levels of ph-AKT-GFP localization without PDGF-A protein addition at the free edge. This is most likely due to low levels of PDGF-A protein, and a more sensitive sensor might reveal intracellular spatial differences in PI3K localization.

We were able to control the CIL-dependent dispersion process in NC cells by modulating PDGF-A/PDGFRα signalling. Depletion of PDGF-A/PDGFRα signalling inhibited dispersion. Most importantly, the inhibition of dispersion by PDGFRα depletion could be rescued by co-injection with N-cadherin, thus proving N-cadherin as the downstream target of the PDGFRα cellular response. Further analysis by immunoblotting showed that pharmacological inhibition of the PDGFR/PI3K/AKT axis does indeed lead to a downregulation of N-cadherin. This demonstrates N-cadherin as a regulator of CIL controlled by PI3K/AKT signalling. A remaining question for further studies will be the link between AKT and N-cadherin regulation.

The requirement of N-cadherin for proper NC migration has been shown in chick, mouse, *Xenopus* and zebrafish embryos (Nakagawa and Takeichi, 1998; Xu et al., 2001; Luo et al., 2006; Shoval et al., 2007; Theveneau et al., 2010; Rogers et al., 2013; Scarpa et al., 2015; Broders-Bondon et al., 2016). Here, we show that the regulation of N-cadherin at the pre-migratory NC stages is PDGF-A/PDGFRα dependent and that loss of N-cadherin by depletion of PDGFRα signalling leads to an inhibition of NC migration. Our data suggest that, at these early stages, inhibition of NC migration by PDGFRα depletion is due to N-cadherin-dependent impairment of CIL. Furthermore, they suggest that the PI3K/AKT pathway is the downstream effector of PDGF signalling during NC EMT.

## MATERIALS AND METHODS

### Embryos, microinjections and micromanipulation

Animals were used according to instructions from the Home Office of the United Kingdom, where animal licences are required. *Xenopus laevis* embryos were obtained and staged as described previously (Nieuwkoop and Faber, 1967), and the embryos were injected at the eight- to 16-cell stage as previously described (Carmona-Fontaine et al., 2008b). Explants were dissected at stage 17 for *in vitro* experiments and plated on a fibronectin-coated dish using 10 or 50 μg/ml fibronectin (Sigma-Aldrich) for plastic or glass dishes, respectively, in DFA medium as described previously (Theveneau et al., 2010). For *in vivo* experiments, Fluorescein-Dextran (3 μg, D1821, Invitrogen) or Rhodamine-Dextran (3 μg, D1824, Invitrogen) were used as tracers, and embryos were fixed at stage 24 to perform *twist* *in situ* hybridization. Embryos were treated from stage 14/15 to stage 24 with small molecule inhibitors, and equal amounts of DMSO were used as controls.

### Single-cell migration, chemotaxis, cell dispersion and CIL

Cell dissociation was performed by incubating in Ca^2+^Mg^2+^-free DFA for 3-5 min before transferring to normal DFA medium. Cells were tracked using the ImageJ Manual Tracking plugin (http://rsb.info.nih.gov/ij). Track speed and persistence were determined using the ImageJ Chemotaxis Tool plugin. Chemotaxis assay was performed as described previously (Theveneau et al., 2010). Heparin-acrylic beads (H5263, Sigma-Aldrich) were incubated overnight at 4°C in a 1 mg/ml SDF1 or 1 mg/ml PDGF-AA (AF-100-13A, PeproTech) solution in PBS. To measure dispersion, NC cells from embryos injected with H2B-mCherry (Carmona-Fontaine et al., 2008b) were imaged for 12 h, and nuclei triangulation was analysed using the ImageJ Delaunay Triangulation plugin (Carmona-Fontaine et al., 2011). For small molecule inhibitor treatment, inhibitors were incubated 1 h before addition of PDGF-A protein (50 ng/ml, PeproTech). To study CIL, an explant confrontation assay was performed as described by Carmona-Fontaine et al. (2008a). For the single-cell confrontation assay, single-cell CIL time was measured from the first frame of contact (t=0) until the last frame of contact (t=end). Protrusion area was analysed as previously described (Law et al., 2013).

### RNAs, morpholinos and inhibitors

PDGF-A MO (8 ng, 5′-AGAATCCAAGCCCAGATCCTCATTG-3′) (Nagel et al., 2004) and the newly designed PDGFRα MO (16 ng, 5′-TGCCCTCATGGCAGGCATCATGGAC-3′) were obtained from Gene Tools. Mouse mRNA mismatches are underlined. Plasmids were linearized and mRNA transcribed using mMessenger mMachine Transcription Kits (Thermo Fisher Scientific). The mRNA constructs injected were membrane GFP (300 pg), nuclear RFP (300 pg, H2B-RFP), PDGFRαw37 (300 pg) (Ataliotis et al., 1995), ph-AKT-GFP (500 pg) (Montero et al., 2003), PI3K-p110CAAX (300 pg) (Montero et al., 2003), mouse PDGF-A (200 pg) (Fruttiger et al., 1999), mouse PDGFRα (300 pg, IMAGE ID 5704645) and N-cadherin (300 pg) (Scarpa et al., 2015). For mouse PDGF-A mRNA transcription, mouse PDGF-A pGEM-1 (Fruttiger et al., 1999) was linearized with *Pvu*II (Promega) and transcribed with SP6 polymerase (Promega). For mouse transcription PDGFRα I.M.A.G.E. Consortium Vector was linearized with *Pac*I (Promega) and transcribed using T7 polymerase (Promega). Pharmacological inhibitors were all solubilized in DMSO (Sigma-Aldrich) and appropriate DMSO controls were used for all experiments. AG1296 (*in vivo* 20 μM, 658551, Merck Millipore), LY294002 (*in vivo* 40 μM, *in vitro* 5 μM, 9901, Cell Signaling Technology), MK-2206 (*in vivo* 100 μM, *in vitro* 5 μM, 1684, Axon Medchem), UO126 (*in vivo* 100 μM, *in vitro* 25 μM, 9903, Cell Signaling Technology).

### Semi-quantitative RT-PCR

RNAs were extracted from NC or ventral non-NC tissue using an RNeasy Mini Kit (Qiagen). cDNA were reverse transcribed using an ImProm-II Reverse Transcription System (Promega). PCR cycles were analysed in pilot experiments. For a primer list and annealing temperatures see Table S1.

### Western blotting, *in situ* hybridization and immunostaining

For immunoblotting, NC cells were lysed (25 cells/lane) in a lysis buffer containing 100 mM Tris-HCl (pH 8.0), 1% Triton X-100, 0.01% SDS, cOmplete Mini Protease Inhibitor Cocktail (Roche) and PhosSTOP Phosphatase Inhibitor Cocktail Tablets (Roche). Protein fractions were isolated by centrifugation (19,500 ***g***, 4°C) in two rounds for whole embryo lysates. NC lysates were applied to SDS gels without the purification step. Protein lysates were analysed by SDS-PAGE using 4-12% NuPAGE Bis-Tris gels (Invitrogen), and subsequently transferred onto Invitrolon polyvinylidene difluoride membranes (Invitrogen). Membranes were blocked with 5% nonfat dry milk and 0.1% Tween-20 in PBS for 1 h at room temperature, before being probed with the primary antibody by overnight incubation at 4°C, followed by incubation for 1 h at room temperature with a horseradish peroxidase-linked secondary antibody (sc-2030; 1:3000; Santa Cruz Biotechnology) and detection using an ECL reagent (Luminata Forte Western HRP Substrate, Millipore). Band intensity was measured by scanning films and analysing by densitometry using ImageJ. *In situ* hybridization was performed as described (Harland, 1991). NC was labelled with digoxigenin-labelled RNA probes against *slug* (Mayor et al., 1995) or *twist* (Hopwood et al., 1989) in *Xenopus*. Immunostaining was performed according to standard procedures (Carmona-Fontaine et al., 2008b). The following antibodies were used: PDGFRα (1:2000, 3164, Cell Signaling Technology), pAKT Ser437 (1:2000, 9271, Cell Signaling Technology), total (pan) AKT (1:2000, 4691, Cell Signaling Technology), GAPDH-HRP (1:2000, sc20357, Santa Cruz Biotechnology), N-cadherin (western blotting 380 ng/ml, immunostaining 6 μg/ml, MNCD-2, DHSB) and E-cadherin (western blotting 60 ng/ml, immunostaining 120 μg/ml, 5D3, DHSB). If required, DAPI was applied with the secondary antibody (20 μg/ml, D9542, Sigma-Aldrich).

### Statistical analysis

Significant differences between two data sets were determined using contingency tables as previously described (Carmona-Fontaine et al., 2008b) [the null hypothesis was rejected if T>3.841 (α=0.05), T>6.635 (α=0.01) or T>10.83 (α=0.001)]. Data sets (western blot data) were analysed as follows: normality was evaluated by the Kolmogorov–Smirnov test and d'Agostino–Pearson test; data sets were treated as normal distributed if found so by the two tests. Normal distributed data were compared using Student's *t*-test (two-tailed, unequal variances) or one-way ANOVA with a Dunnett's multiple comparisons post-test. Data sets that were found not to follow a normal distribution were compared using Mann–Whitney's test or a nonparametric ANOVA in Prism 5 (GraphPad). Normalized western blot data were analysed by one-way ANOVA followed by Student Newman–Keuls test for multiple comparison differences. All analyses were performed in Prism 5 (GraphPad).
